# The Teaching of Temperance

**Published:** 1909-07-10

**Authors:** 


### THE TEACHING OF TEMPERANCE.

We have received a copy of the syllabus of "Lessons on Temperance for Scholars attending Public Elementary Schools," issued by the Board of Education as a model syllabus, to which all teaching on this subject in elementary schools should conform in principle and, indeed, so far as possible also in detail. The syllabus is divided into three sections, of which the third is exclusively designed for children over the age of twelve, and consists in essence of a description of the evil consequences of alcoholic excess. The first section deals mainly with food and drink in general, and the second is given up to alcoholic beverages and their effects. The whole syllabus is to be covered in three lessons at the most.

Now the inclusion of elementary hygiene in the teaching of elementary schools is obviously commendable, and it is right that this should include the elementary facts of the physiology of food and drink. But in this syllabus the term temperance clearly refers throughout to alcoholic beverages and not to food and drink in general. This, we venture to think, is a mistake. The great principle to inculcate is temperance in all things, the greater including the less. There are, however, in the syllabus certain "notes," intended to provide the teacher with a sound basis of scientific fact and principle upon which to found his instructions. We regret that these statements, in our opinion, are open to serious criticism. They convict their compiler of some measure at least of bias and inaccuracy and confusion of thought, scarcely appropriate to the preparation of an authoritative official syllabus.

In the first place pure alcohol is confused throughout with alcoholic beverages, and although the effects of alcoholic excess are described in detail, the broad fact that throughout the centuries the great majority of mankind have drunk alcoholic beverages without ill-effect is entirely withheld. No attempt is made to explain to the child why civilised communities have continued to find pleasure and profit in the moderate consumption of alcoholic beverages, and the fact is withheld from them that in the preparation of many beverages prized by mankind for their taste, thirst-quenching, and nutritional properties, alcohol is essentially a by-product rather than an active ingredient. The alcoholic content of ginger-beer is overlooked, as is the fact that the Excise authorities for the purposes of inland revenue ignore the alcoholic nature of a beverage which contains less than 2 per cent. of proof-spirit.

To turn from errors of omission to those of commission we cannot agree that bottled beer contains on an average 7 per cent. by volume of alcohol, and that Lager beer usually contains as much as 4 per cent. We entirely disagree with the remarks made concerning the nutritive value of good beer. The Hospital has this year dealt exhaustively with the whole question of beers, and published the results of close investigations into the chemical composition and dietetic uses of malt liquors; and the conclusions derived from the report of The Hospital Commission are entirely at variance with the dicta of the Board of Education syllabus. Good beer under suitable circumstances is peculiarly well adapted to the quenching of thirst, and fulfils accurately the physiological requirements of the thirsting body. The bold dogmatic statement repeatedly made that water is the best fluid to drink surely demands some qualification and explanation. If the child is told that the best drink is water, some rough directions at least should be supplied for the discrimination between good water and bad water. The assumption is that any water is drinkable. We have the true cause of temperance at heart, but we must repeat once more that enduring temperance teaching can only rest upon a solid foundation of scientific accuracy. When the child finds that certain of the statements he has been dogmatically taught will not stand the test of adult experience and reflection, there is danger that the many truths contained in this syllabus will be overshadowed and discredited and will bear no fruit. The Board of Education cannot be congratulated upon its syllabus for the teaching of temperance; but we still hope that those who are obliged to use it for the instruction of their pupils will bring to bear upon it all their common sense and impartiality and knowledge of the world, and thus learn to discriminate between its wheat and tares.

